# Neuro-ophthalmic semiology in Jacometto Veneziano’s Portrait of a Lady (1470 s)

**DOI:** 10.1007/s00415-026-13623-5

**Published:** 2026-01-16

**Authors:** Hutan Ashrafian

**Affiliations:** Institute of Global Health Innovation, The Department of Surgery and Cancer, Imperial College London, 10th Floor Queen Elizabeth the Queen Mother (QEQM) Building, St Mary’s Hospital, Praed Street, London, W2 1NY UK

**Keywords:** Neuro-ophthalmology, Ptosis, Mydriasis, Oculomotor nerve, Goitre, Systemic lupus erythematosus

The *Portrait of a Lady*, attributed to the Venetian Renaissance painter Jacometto Veneziano (active in Venice from the early 1470s to the late 1490s), is a small oil-on-panel portrait dated to the 1470s. The sitter has sometimes been interpreted as a courtesan or sex worker on the basis of costume and social semiotics (for example, the yellow scarf and dress conventions), although this remains an attributional inference rather than a clinical datum. The image is notable for intense centrofacial erythema over the nose and cheeks with relative suborbital/periorbital sparing, a receding hairline with wispy red hair suggestive of alopecia, possible perimandibular fullness (raising the possibility of parotid enlargement), a swollen right upper eyelid (compatible with periorbital edema or lacrimal gland involvement), a conspicuous goiter with a heavier-set habitus, thin eyebrows with reduced lateral third density, and erythema of the auricle and nasal tip. These visible findings have previously been framed in a differential [1] centered on systemic lupus erythematosus (SLE), potentially with secondary Sjögren’s syndrome and/or coexisting autoimmune thyroid disease, with rosacea considered but viewed as less morphologically congruent, secondary syphilis raised but discounted on historical chronology (the widely recognized epidemic emergence in Italy occurring in the 1490 s), and relapsing polychondritis discussed as a lupus-associated possibility given auricular erythema (See Fig. 1).

**Fig. 1:**
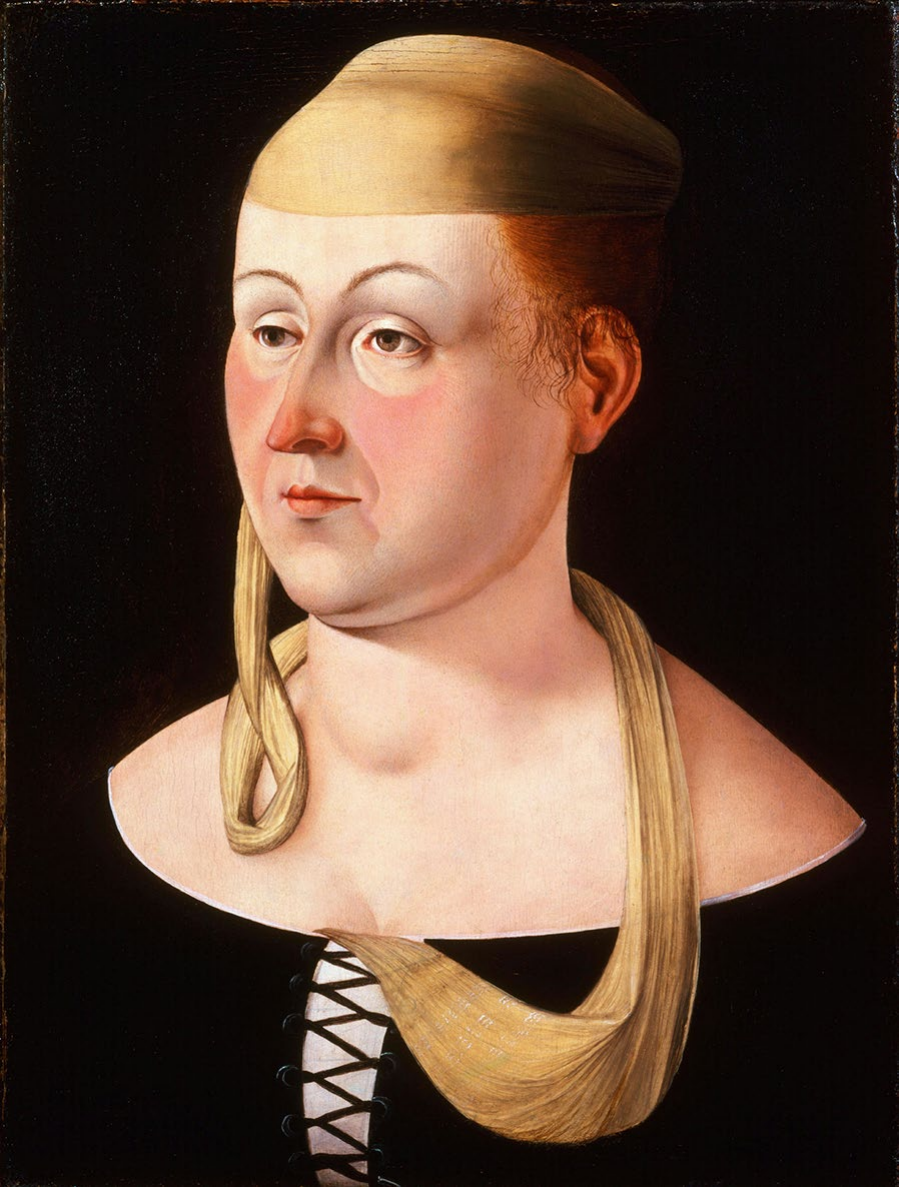
Portrait of a Lady (c. 1470 s), attributed to Jacometto Veneziano. Recto. Oil on panel. Philadelphia Museum of Art, John G. Johnson Collection, 1917 (Cat. 243), © Philadelphia Museum of Art (the image reproduced in this manuscript is in the public domain and may be freely used without restriction)

On further examination of the image, additional neuro-ophthalmic signs are apparent. These include mild bilateral ptosis, bilateral pupillary dilation (mydriasis) with possible subtle anisocoria (R ≥ L), and possible reduction of frontalis recruitment (“smooth” forehead). These findings suggest a canonical neuro-ophthalmic pattern that localizes to oculomotor parasympathetic involvement and a distinct differential framework.

SLE can, albeit rarely, manifest as inflammatory cranial neuropathy including oculomotor dysfunction (ptosis, diplopia) and reported pupillary abnormalities [2, 3]. In addition, a “lupus-adjacent” diagnosis could be thrombotic/vasculopathic disease within an autoimmune origin, most plausibly antiphospholipid-spectrum (APS) microvascular or macrovascular events presenting with ptosis and oculomotor palsy even when full systemic criteria for SLE and APS are incomplete [2, 3]. Furthermore, bilaterally enlarged pupils suggest a postganglionic parasympathetic/autonomic disorder within the tonic pupil spectrum toward bilateral parasympathetic or immune-mediated cranial neuropathy integrated with the cutaneous (lupus spectrum immune/inflammatory dermatosis) or endocrine (thyroid) phenotype. Finally for completeness, a less likely possibility includes a subtle anisocoria (R ≥ L) and the left globe is subtly supraducted that could accommodate compressive third-nerve palsy.

Jacometto Veneziano worked in a Venetian portrait tradition that prized fine-grained verisimilitude in skin tone, eyelid contour, hairline, and the subtle asymmetries of gaze and expression, producing images that can function, in effect, as high-resolution clinical vignettes. In that sense, the Venetian Renaissance, no less than the better-canonized Florentine and Roman schools, offers a parallel archive of embodied human biology, allowing Venetian painting to contribute to our understanding of physiology and frailty of lived experience across five centuries.

## Data Availability

Data sharing is not applicable to this article as no datasets were generated or analyzed during the current study.
